# A National Dental Museum

**Published:** 1895-09

**Authors:** H. B. Noble

**Affiliations:** Washington, D. C.


### A NATIONAL DENTAL MUSEUM.[1]

BY DR. H. B. NOBLE, WASHINGTON, D. C.

Some years since I claimed exemption from jury duty as a surgeon, and after a legal battle was sustained in this position, and no dentist has since then been placed in the jury-box.

Many cases in actual practice have sustained this position. A case in point: The jaw of Hon. William H. Seward, the Secretary of State under President Lincoln, was broken in his attempted assassination. A dentist of New York (Dr. Gunning) was called upon to take charge, and did take charge, at the request of the Surgeon-General of the United States, and conducted the case to a complete restoration, and to the satisfaction of every one.

If we are dental surgeons and a specialty of the medical profession, let us take our proper place in the National Medical Museum and Library, unfortunately so little known, and slightly appreciated by the dental profession throughout the country.

Here we have in a fire-proof building a valuable collection of specimens and books, under the efficient management and fostering care of Dr. John S. Billings, and maintained at government expense. It is a part of, and on a par with, the other scientific departments of government.

Here should be found a history of dentistry and a general depository for dental archives of all things pertaining to it which may have a present or a future historical or educational value.

That the profession is imbued with the spirit of homage to those whose labors and achievements have elevated our calling to a place among the liberal professions was made manifest at the late gathering in Philadelphia to honor the name of Horace Wells, the discoverer of "anæsthesia."

Could there be a more appropriate place for the repose of memories, whether of marble, bronze, or painted canvas, or one's own laboriously collected library?

Such a museum or library would be an appropriate place for the proposed statue of Dr. Horace Wells, surrounded by a library such as that of Dr. H. J. McKellops, of St. Louis, and the historical collection of Dr. Taft's committee of the World's Columbian Dental Congress.

---

[1] Read before the Massachusetts Dental Society at their annual meeting, June 5 and 6, 1895.

Here should be recorded all the great and important inventions and scientific discoveries that have been and are being made, and the names and record of eminent and celebrated men who have labored for the advancement and elevation of our profession. Many of these records are now in the keeping of local societies and individuals, and if not soon cared for will be lost to the general profession.

Such a museum, library, and memorial hall, in connection with the National Medical Museum, would receive the aid and fostering care of the general government, and be under its care and protection.

If we work in harmony with the medical profession, such a hall and library of our own can be secured.

